# How useful do communities find the health and wellness centres? A qualitative assessment of India’s new policy for primary health care

**DOI:** 10.1186/s12875-024-02343-2

**Published:** 2024-03-19

**Authors:** Shriyuta Abhishek, Samir Garg, Vikash Ranjan Keshri

**Affiliations:** State Health Resource Centre, Chhattisgarh, Raipur India

**Keywords:** Primary health care, Health and wellness centres, Community perception, Universal health care, India

## Abstract

**Background:**

The policy attention to primary health care has seen a global upswing in recent years, including in India. Earlier assessments had shown that a very small proportion of Indian population used the government primary health facilities. Starting in 2018, Indian government has established more than 100,000 Health and Wellness Centres (HWCs) to increase rural population’s access to primary health care. It is crucial to know how useful people find the services of HWCs.

**Methods:**

A qualitative inquiry was made to understand the perceptions, experiences and expectations of the rural communities regarding HWCs in Chhattisgarh state. Fourteen focus group discussions were conducted with community members. The study areas were chosen to include both the central and remote districts of the state. The study used accessibility, availability, acceptability and quality (AAAQ) framework to assess HWCs.

**Results:**

Community members felt that the most important change brought about by HWCs was to offer a wider range of curative services than previously available. Services for noncommunicable diseases such as hypertension and diabetes were seen as a key value addition of HWCs. People felt improvements in services for acute ailments also. The services people found missing in HWCs were for injuries, dental care and mental health. In people’s experience, the availability of essential medicines and point-of-care tests at HWCs was satisfactory and the treatment was effective. People appreciated the supportive behaviour of health workers in HWCs. They did not find the referrals from HWCs as excessive but often faced difficulties in receiving necessary services at higher facilities.

**Conclusions:**

The assessment based on community perceptions showed that the services of HWCs matched well with people’s needs of curative primary care. It shows that people are willing to use the government facilities for primary health care if the services are relevant, adequately functional and accessible.

**Supplementary Information:**

The online version contains supplementary material available at 10.1186/s12875-024-02343-2.

## Background

The primary health care (PHC) approach is the foundation for building equitable and effective health systems that can address the health needs of people throughout their lives [1, 2]. In 2018, countries across the globe renewed their commitment to implement PHC through the Astana declaration [3]. PHC has been agreed as the desired path to achieve the goal of Universal Health Coverage (UHC) [4]. This approach due to its strengths in cost-effectiveness and equity is especially relevant to needs of health systems in the low- to middle-income countries (LMICs) such as India, which has a large under-served population [5].

In India, the introduction of Health and Wellness Centres (HWCs) in 2018 was seen as a major step forward from provisioning of ‘selective’ to ‘comprehensive’ primary health care spanning preventive, promotive, curative, rehabilitative and palliative care with a focus on continuum-of-care [6]. HWCs have been developed by reconstituting the existing primary facilities to deliver comprehensive primary health care [6]. Prior to HWCs, India had two kinds of public primary care facilities: a Sub Health Centre (SHC) catering to around 5000 population and a Primary Health Centre for 30,000 population. Each SHC was staffed with an Auxilliary Nurse cum Midwife (ANM) and a paramedic and provided child immunization and primary services limited to reproductive health [5, 7]. Primary Health Centres (PHCs) provided services largely focused on communicable diseases and reproductive health [7]. HWCs are expected to provide preventive, promotive, rehabilitative and curative care for reproductive and child health, communicable diseases, noncommunicable diseases (NCDs), eye ailments, ear-nose-throat ailments, dental health, mental health, elderly health, palliative needs, acute medical conditions and injuries [6]. The national target of operationalising 115,000 HWCs by upgrading SHCs was met by 2023 [8].

The health workforce at each HWC has been visualised as a primary care team [7]. It includes a Community Health Officer (CHO) who is a nurse or Ayurvedic practitioner with additional training in primary health care. The CHO is envisioned as the leader of HWC team. Other members of the team are two to three paramedics including at least one ANM. In addition, government funded community health workers known as Accredited Social Health Activists (ASHAs) act as its extended team, linking communities to HWC facility [6].

HWCs aim to ensure a people-centered, holistic and equity sensitive response to population health needs [9–11]. Assessments in India prior to the inception of HWCs had often concluded that a very small proportion of population used the government primary care facilities [10, 12]. Thus, the success of HWCs is dependent on how people perceive and utilize their services. However, there is no evidence available so far on this. Hence, in this study, we explored the perceptions of community members about HWCs.

## Methods

### Study design

We conducted a qualitative inquiry to understand the perceptions of the community, their experiences and expectations regarding HWCs.

### Study setting

The study was conducted in rural areas of Chhattisgarh state. Chhattisgarh was one of first states in India to implement a pilot on HWCs. It is one of the poorer states in India and 77% of its population lives in rural areas [13, 14]. Around a third of the rural population in the state belongs to indigenous communities, officially known as the scheduled tribes, one of the most vulnerable social groups in India [14].

### Sampling and data collection

This study was limited to rural communities covered by the HWCs in operation for more than a year after being upgraded from SHCs, with appointment of a CHO. The data collection took place during September 2023. At the time of data collection, Chhattisgarh had operationalised 3370 HWCs at the SHC level i.e., a CHO had got posted in each of those [8]. Out of these, 2650 HWCs had completed a year or more in operation after a CHO getting placed [8]. All the CHOs in Chhattisgarh are nurse graduates who had received the stipulated training to work in HWCs. In addition to a CHO and two to three paramedics, each HWC in Chhattisgarh has around twelve ASHA CHWs who are locally known as ‘Mitanin’.

The study was conducted in three districts of the state. Two districts; Kanker and Kondagaon were in a remote and forested region and have predominantly scheduled tribes population. The third district was Raipur that lies in the central part of Chhattisgarh and has predominantly non-tribal population [15]. These districts were chosen to include both the central and remote districts of Chhattisgarh.

For the purpose of this study, eleven HWCs were selected. Of the selected HWCs, six were in Raipur district, two in Kondagaon district and three in Kanker district. The average population covered by the above HWCs was around 4700. The average number of outpatients who visited a HWC was around 360 per month. Each centre had a CHO posted there. Nine of the CHOs were female. The HWCs in Raipur district had two paramedics each, including an ANM. The HWCs in Kanker and Kondagaon had two ANMs each and three HWCs also had another paramedic. All except one HWC had a government building. The buildings had been freshly painted when they became HWCs. Nine of the eleven HWCs had a ramp. All except one HWCs had the necessary furniture, including the labour table. In all the HWCs, medicines were available for primary care of hypertension, diabetes, simple acute ailments, minor injuries and ante-natal care. Each HWC had a functioning apparatus for blood pressure measurement. All HWCs had the point-of-care test for blood sugar in addition to tests used for ante-natal care.

Focus group discussions (FGDs) were conducted with community members. A guide was developed for the FGDs (Additional File S1). The main points around which the FGDs were structured were – what people found useful about the services of HWCs and what they found lacking.

A total of fourteen FGDs were conducted. Eight FGDs were in Raipur district and six in Kanker and Kondagaon districts. The FGDs covered the villages in the catchment population of HWCs. Eleven of the FGDs were in villages close to a HWC and three FGDs were in communities which were relatively distant. The FGDs were conducted in two kinds of locations - in the communities and at HWCs. Eight of the FGDs took place in HWC buildings where the participants were selected out of patients and their attendants visiting the HWC. Six FGDs were conducted in the communities, at house of one of the participating community members. The participants for FGDs were selected based on age, gender, disability and caste to ensure maximum variation among the participants. The local Mitanin CHWs helped the researcher in finding the diverse kinds of participants and in requesting them to join the FGD. Efforts were made to build a rapport with the participant community members before initiating the FGDs. Each FGD included five to seven participants and a total of 75 individuals participated. The average duration of the FGDs was 40 min. The shortest FGD lasted 35 min and the longest took 50 min. The audio from FGDs were recorded after taking written informed consent from the participants. Ethics approval for the study was obtained from the Institutional Ethics Committee of the State Health Resource Centre, Chhattisgarh. The COREQ checklist for transparency in reporting qualitative research is enclosed (Additional File S2)

### Data analysis

The FGD recordings were transcribed verbatim in Hindi. The transcriptions were checked for completeness and consistency and thereafter translated to English. The translated transcripts were checked for completeness and consistency by comparing it against the transcriptions. Three out of the fourteen translated transcripts were back-translated to the Hindi by an independent translator for validation. Reading and re-reading of the translated transcripts were done for data familiarization. The important information was extracted from the description. and then coding was done line by line. During analysis, the codes were clustered under a-priori themes and emergent themes and sub-themes [16].

For the thematic analysis, we used a framework comprising of four dimensions - accessibility, availability, acceptability and quality (AAAQ). The importance of these dimensions in healthcare has been agreed by the United Nations [17, 18]. The above dimensions along with affordability of care have been recognised by researchers as what constitutes meaningful access to healthcare [19].

Lastly, to ensure that the study findings reflect and represent the data provided by participants, peer debriefing was done.

## Results

The socio-demographic characteristics of community members who participated in the study are given in Table 1.

**Table 1:** Socio-demographic profile of community participants

Characteristic	Category	Frequency (%) *n* = 75
Age	Under 30 years	10 (13.3)
	30–45 years	17 (22.7)
	46–59 years	22 (29.3)
	60 years or above	26 (34.7)
Gender	Male	22 (29.3)
	Female	53 (70.7)
Social group (Caste category)	Scheduled Castes	13 (17.3)
	Scheduled Tribes	16 (21.3)
	Other Backward Classes	35 (46.7)
	Others	11 (14.7)

The findings from the FGDs with community members are presented in the following sub-sections.

### Accessibility

#### Physical distance of HWCs from the communities

Community members appreciated that the healthcare services of HWCs were available close to them and easy to reach.

*It is a government health centre that is near our house, and it is very convenient to visit. We walk just thirty steps and we reach this centre. It saves us time, as compared to going to a hospital which is not nearby, government or private.* (56-year-old, female, Kanker district)

The close location of HWCs was also convenient for the families of patients, especially for services related to childbirth.

#### Hours HWCs stay open for service delivery

Community members also appreciated that the services were available daily (except government holidays) and throughout the day hours in HWCs. The patients could contact the CHOs over phone. This reduced the uncertainty for patients when a CHO was not present at HWC and it increased their convenience in using the services of HWCs.

*This health centre is open for most hours in the day. We can walk-in anytime. Sometimes the staff is not there but that doesn’t happen a lot. When they are not available, we can call them on phone. I have the phone number of the CHO.* (49-year-old, male, Raipur district)

### Accessibility for people living farther from HWCs

For people living in hamlets farther from HWC, it was important that a CHO visited their village. The community visit of a CHO was usually once or twice in a month. They took treatment from CHO during such visits. When they needed care on other days, they visited the HWC.

*Whenever there is a session (CHO visit) here, I attend it. Usually, it is on the village immunisation day. I take medicine from here. Other times, I go to the HWC (in nearest village). The CHO gives me the medicine.* (25-year-old, female, Kanker district).

Though the distance of a HWC was a lot less than the nearest PHC, it was not easy for people residing in villages farther from the HWC to access it, especially for an acute need. Some people in such remote locations preferred to take treatment from the informal providers available closer.

*We cannot wait till she (CHO) visits our village. The HWC is not nearby. If there is any urgency, we go to the informal private practitioner for immediate treatment which is an injection or bottle (intravenous drip).* (50-year-old, female, Kanker district)

### Availability

#### The range of curative services made available by HWCs

According to community members, a key change brought about by the upgradation of SHC to HWC was that a wide range of curative services which were previously not there had now become available.

*The building of HWC is not new. It has been there for many years. But many types of treatment are now being provided there.* (58-year-old, male, Kanker district)

The community members found the services of HWCs suitable for needs of large sections of population especially women, children and elderly.

#### Availability of NCD care in HWCs

A majority of the community members felt that they had benefitted from the availability of services for NCDs at HWCs. They emphasized the availability of screening services and follow up for hypertension and diabetes cases. Earlier blood pressure was measured only for pregnant women during their ante-natal care, but now anyone could walk-in to get their blood pressure measured. Those thirty years or older were screened for hypertension and diabetes. Some community members shared that their blood pressure had been initially checked by a Mitanin and she had advised them to visit a HWC for diagnosis and treatment.

*Our Mitanin (ASHA) checked my blood pressure (BP) and informed me that it was high. She asked me to visit the HWC. The CHO there told me that I have high-BP disease and need to take medicines. Since then, I have been taking the medicine from there every month. I visit the HWC every fortnight or once a month to get my BP checked.* (60-year-old, female, Kondagaon district)

The role of HWCs in identification of chronic diseases was highly appreciated by the community members. The regular availability of medicines for diseases such as hypertension and diabetes was also very important to them.

*My BP was as high as 150 when I first went there (HWC). I was shocked because I had never experienced any problem with my body. But then they (CHO) gave me a good medicine. I take it from here every month, and it has been fine ever since. Medicines are usually available here. Only once it was not available here. They have told me to visit a week before my medicines get over, so that they can ensure that I get the refill in time.* (48-year-old, female, Raipur district)

Some community members got their check-ups done at HWC even when they were taking medicines or treatment from other providers. Some participants did not find the medicine for hypertension suitable initially but were later considering going back to treatment at the HWC as alternative treatments were not effective in controlling the high BP.

*Medicine which was given at HWC didn’t suit me for high BP as I felt it was giving me dizziness. I shifted to taking ayurvedic medicine because it had fewer side effects. I took medicines from a private ayurvedic doctor. But I continued get my BP checked by the CHO each month when she visited our village. Now I am thinking of going back to the HWC medication again because the CHO checked my BP and told me that it was out of control.* (61-year-old, male, Kanker district)

Some of the participants had found taking medicines for diabetes from the private sector unaffordable and they switched to the treatment at a HWC after visiting it for a blood sugar test. In addition to the medicines, people valued the role HWCs played in providing check-ups as follow-up for chronic diseases.

*I have sugar (diabetes) for a long time now. I used to take medicine from a private clinic. It used to be very expensive. But now I take it from the HWC. The medicine HWC gives is equally good and I get it for free. I used to visit the HWC for getting the test (blood sugar test) done anyway.* (55-year-old, male, Kondagaon district)

Community members expressed their satisfaction with services at HWCs for some other chronic problems such as allergies. An adolescent participant appreciated that diagnostics such as the haemoglobin test had now become available for them at HWCs, and that they were not simply handed-over medicines to improve their iron-levels.

One of the community members shared that her daughter recently had been diagnosed with sickle cell disease upon referral made by the CHO to a secondary facility based on their family history. In this case, the family had to go to a distant public hospital to get the diagnosis for sickle cell disease. The HWC could only provide a referral but had no diagnostic services for sickle cell disease.

There were several NCDs for which people wanted services but which were not available yet in HWCs. Community members noted that though the care for hypertension and diabetes had improved, there were other diseases such as epilepsy, respiratory problems, mental health problems and sickle-cell disease that needed to improve. They would have preferred to seek treatment from a HWC if it where available there.

*My brother has the disease that leads to seizures and we have to take him to a private hospital for treatment because there was no proper treatment available for him at the HWC.* (32-year-old, male, Kondagaon district)

#### Availability of services for needs other than NCDs

A majority of community members particularly the adolescent girls, women and elderly people shared that HWCs were their first point of contact in case of an acute illness.

*I was informed by a staff at our school that we could come here (HWC) if we get injured while playing etc. I have come here to get tetanus toxoid injection with my friend.* (14-year-old, female, Kondagaon district)

People were satisfied that the treatment and medicines for acute ailments were available in HWCs.

*For cold, cough, body pain, fever etc. I visit this centre (HWC). They give medicines for all these problems. I had itching on my arms and small boils. They gave me an ointment for that and also some tablets. The itching reduced and boils are gone too.* (30-year-old, female, Kondagaon district)

One of the community members expressed her trust in the HWC staff for treatment of her young child. She also appreciated that the HWC guided her when a referral was needed.

*I bring my toddler here when he has cough. He gets cough and cold frequently. Their medicine helps. They also advised me to take my child to another hospital when his fever was not going down. They kept in touch with us throughout the course of illness* (24-year-old, female, Raipur district).

The pregnant women shared that they visited HWCs routinely, often on the advice of the Mitanin. People felt that the availability of maternal and child health services had improved in HWCs as now a CHO was available in addition to the paramedic staff present earlier. They particularly found a big improvement when the CHO resided close to the HWC premises.

*I had an untimely labour pain during the seventh month of pregnancy, and I was brought here. We called the CHO, she came immediately. The CHO lives here only. I had a premature child but he is doing better now.* (26-year-old, female, Raipur district)

The people felt that many emergency services such as the treatment for injuries requiring suture were not available in HWCs.

*I had met with an accident last month and I came here for first-aid. But then the doctor said stitches may be needed so I was taken to the district hospital.* (31-year-old, male, Kanker district)

People were yet to gain confidence in HWC services with respect to emergency care as the services were at a preliminary stage and limited in range.

*If there is an accident, we prefer to go to a private hospital. I am not sure if the CHO here can provide all the treatment.* (32-year-old, male, Raipur district)

The access to emergency care was further limited during the evening and night hours where none of the staff lived close to HWC premises.

*I would not visit the HWC for an emergency as it is closed at evening. There is no staff living here. They commute from the city.* (32-year-old, male, Raipur district)

The community members felt that curative care for dental problems was an important need but HWCs did not offer that.

*I had severe tooth pain recently. If dental care was available at HWC, it would have been good. We wouldn’t have to go to private clinics, as they are not cheap. We can’t afford so we ignore the problem. The CHO here told me to go to the bigger hospital (CHC) but it’s not nearby. A dental surgeon from the hospital should visit here instead.* (60-year-old, male, Raipur district)

### Acceptability

#### Approachability of HWC staff

Most people emphasized that they found the HWC staff very approachable. They felt that the behaviour and attitude of the team towards the community was positive. They shared that they found it easy to communicate with HWC staff including the CHO.

*The CHO here is like our family. We can talk to her anytime. We can easily come to the HWC as it is nearby. She (CHO) treats us well.* (60-year-old, female, Kondagaon district)

People could contact the HWC staff over phone and it showed that they were easy to approach. They felt that CHOs had a caring attitude towards them.

*I can call them if I have any problem, both the ANM and the CHO. When I had diarrhoea, I called the CHO. She had sent the Mitanin with medicine (oral rehydration salt sachets). It feels good to have someone like that.* (55-year-old, female, Kanker district)

#### Responsiveness of HWC staff including the CHOs

The community members, especially the elderly, appreciated that the CHOs listened to their health problems and tried to address those.

*She listens to all our health issues patiently. I am an old person. We always have some or the other health issue. But she tries to address those.* (60-year-old, female, Kondagaon district)

The pregnant women had a positive experience at HWCs as they felt supported by the staff at HWCs throughout the pregnancy and with newborn care.

*I had my delivery in HWC two months back. We didn’t face any problems, as compared to my previous delivery in the district hospital. The staff in big hospitals don’t care about you. You would be lying on the bed and asking for help but they won’t listen. But in the HWC, they give all their support to you. They give all their attention as they don’t have many patients at the same time.* (27-year-old, female, Raipur district)

#### Role of community health workers

The community members appreciated the complementary role played by Mitanins (ASHAs) in facilitating utilization the services of HWCs. The Mitanins helped in screening for diseases or risk factors. Mitanins also followed up with the patients at home, for the chronic diseases as well as acute problems.

#### Unmet expectations regarding some preferred forms of medication for acute ailments

Some people expressed their preference for receiving medication in form of injections, especially for acute problems.

*I wish the CHO provided injections when patients have a lot of pain. One cannot depend on tablets for every problem. Injections can give immediate relief. That is why we sometimes have to go to the informal private practitioner.* (31-year-old, female, Kanker district)

#### What’s in a name?

Hardly any of the participants used the name ‘Health and Wellness Centre’ for HWCs. They still called it a Sub Health Centre or a hospital.

### Quality

#### Effectiveness of treatment provided by HWCs

Most of the community participants were satisfied with the effectiveness of the treatment they received at HWCs. They appreciated that the medications they took from HWCs were effective in addressing their illnesses, including in managing the chronic diseases such as hypertension and diabetes. They were happy to rely on the quality of check-ups and tests at HWCs such as the BP measurement and blood sugar test. However, they were not satisfied with the effectiveness of services for injuries or many other emergencies.

Pregnant women felt that the quality of services for them had improved after the SHC became a HWC. At the same time, pregnant women with history of complications did not find HWC as capable of ensuring a safe delivery for them.

*I get my routine check-up during pregnancy from the HWC. They check my weight, do blood tests and give medicines, and they counsel me about what to eat. But I had a miscarriage last time. So, I am planning my delivery in a private hospital in the city. My family doesn’t want to take any risk this time.* (29-year-old, female, Kanker district)

#### Behaviour of HWC staff

The community members unanimously expressed that the behaviour of CHO and the other HWC staff was good towards the communities and patients. They contrasted it against the behaviour they encountered from some of the medical staff in over-burdened hospitals.

#### Cleanliness in HWCs

The responses varied. While many participants did not find any problems regarding cleanliness in HWCs, a participant expressed disappointment with the lack of cleanliness in the labour room of a HWC.

#### Referral system from HWCs to higher centres

The community participants felt that the CHOs guided them when they were referred to a higher centre.

*I am grateful that by talking to the CHO at the HWC, at least we get some guidance on where to go if treatment is not available there - whom to approach and what is the problem exactly. Earlier we did not have such guidance available.* (69-year-old, male, Raipur district)

The HWC staff also helped the patients requiring secondary inpatient care to get enrolled in the government health insurance scheme. Another positive aspect was that regular check-ups for chronic diseases such as hypertension and diabetes could be accessed at HWCs even when the medication was prescribed by a secondary hospital.

However, there was little communication from HWCs to the concerned higher hospitals regarding each patient referred. Many patients referred by HWCs to higher facilities found incomplete services there.

*My issue is that my BP was not under control but my sugar is fine. So, the CHO sent me to government hospital and there the doctor prescribed me a medicine which was not available there. So, I had to buy it from the private medicine shop. It is expensive for me, but what to do. I am getting my BP and sugar checked at the HWC monthly.* (53-year-old, female, Kondagaon district)

For patients taking treatment from secondary or tertiary hospitals, there was often no coordination from the hospital side with the concerned HWC for future follow-up.

**Fig. 1:**
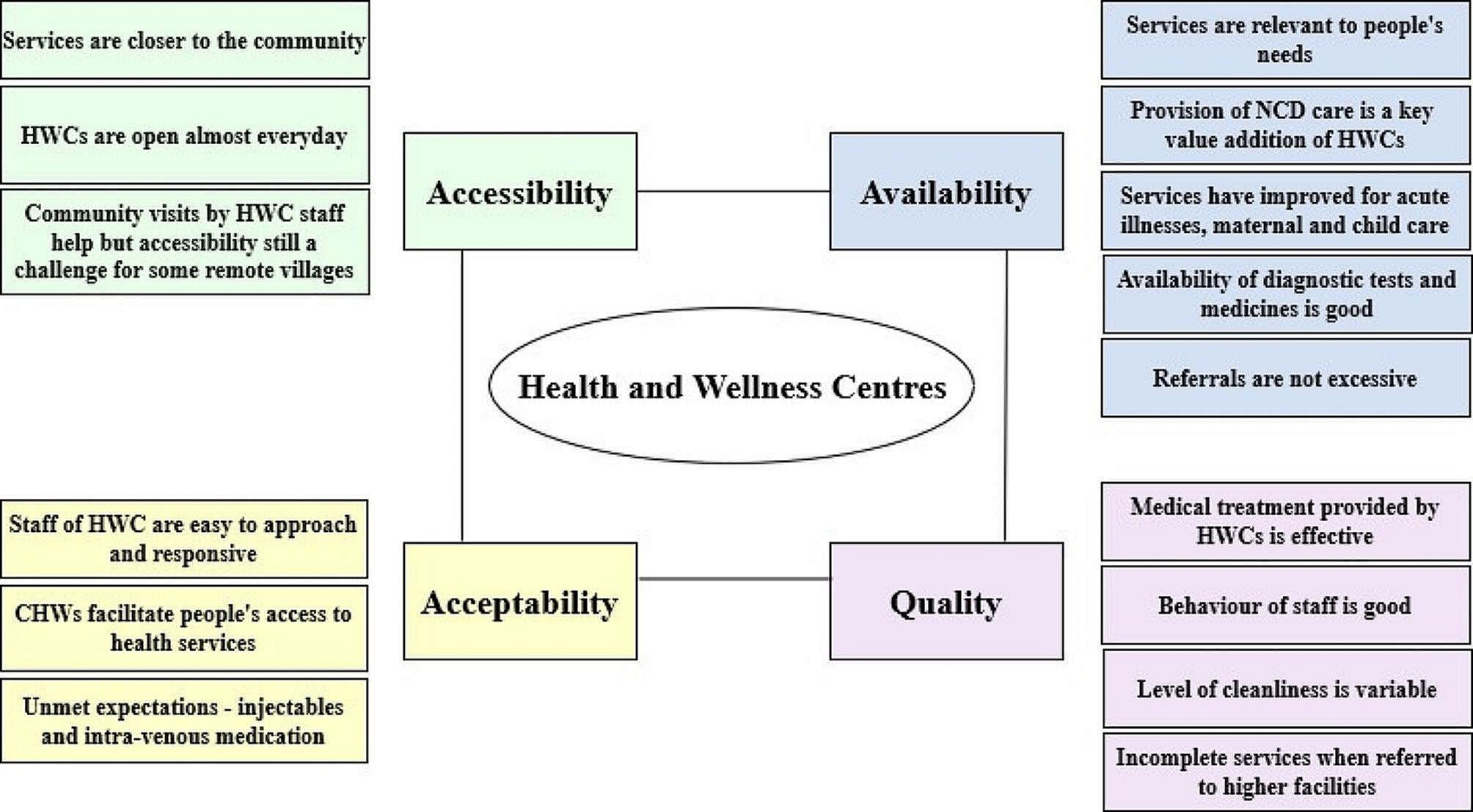
Summary of findings on community perceptions of HWCs

In line with the thematic framework of the study, the key findings have been summarised in Fig. 1.

## Discussion

HWCs are the key policy initiative for strengthening primary health care in India. However, evidence on community perception of HWCs and its services is scarce. This was one of the first studies on the community expectations, experiences and perspectives regarding the services of HWCs. The study used accessibility, availability, acceptability and quality (AAAQ) framework to assess HWCs. The community perception of HWCs showed significant gains for primary health care on all the four dimensions.

HWCs increased accessibility by reducing the distance barrier and having suitable timings. Existing evidence has emphasised that utilisation of primary care facilities can increase if the accessibility can be improved [7, 10]. In addition to the proximity, the accessibility of HWC staff over phone reduced uncertainty for people. People are less likely to utilise a facility if they are not sure if the providers will be present when they reach there. Staff absenteeism has been reported as a critical weakness in Indian PHCs [20, 21]. Our study found that HWCs fared better as community perception on presence of staff was satisfactory.

Our study did not examine the availability of services at HWCs from the supply side. Instead, we focused on the people’s perspective and found it to be multi-dimensional. Most importantly, people found the services of HWCs as relevant to their health needs. Existing studies in India had shown that most people chose providers other than the government facilities for primary care presumably because the services offered were not in tune with their needs [12, 22]. According to the community feedback received in our study, the different essential components of care such as medicines and tests were available in HWCs. Earlier studies have shown that availability of free medicines is important for government facilities to attract enough patients and to meet their treatment needs [23]. People appreciated that HWCs provided screening and follow-up and not just episodic care. This is an important transformation for the public sector system for delivering primary health care in India [24].

The HWC policy aims to provide comprehensive primary health care services [6]. The range of curative services in HWCs showed a significant increase compared to SHCs. This represents a significant advancement as primary care delivered by public sector in India has often been criticised for offering selective primary care [10, 24, 25]. An earlier study had reported that people used the government primary facilities mainly for maternal and child care services [23]. This was because the PHCs and SHCs provided very few services other than those related to maternal and child health [23–25]. A study on evolution of the PHCs in India has shown that their services were shaped by vertical programmes which were selective, top-down and target driven in nature [26]. The outpatient care, which constitutes a large part of people’s curative health needs, was largely neglected and unavailable in government primary facilities [26].

Our findings show that people perceive a crucial value addition of HWCs in making available services for the NCDs. This is an important change as NCDs currently constitute the most significant disease and disability burden globally as well as in India. HWCs were able to provide primary care for hypertension and diabetes. Still there were a few important NCDs such as epilepsy, mental illnesses, sickle cell disease and chronic respiratory diseases for which people’s curative care needs were not getting met from HWCs. There were improvements in availability of maternal care and treatment for communicable diseases. HWCs are also expected to provide care for injuries, dental care and palliative care but these services were largely unavailable. An Indian study on primary care of depression had also recommended the need to strengthen the role of HWCs in providing the necessary services and linkages on mental health [27].

The community feedback received in our study was mainly related to curative care. HWCs were involved in prevention activities for key infectious diseases such as malaria even as SHCs and they continued to so after their upgradation [7]. The delivery of advice for prevention of NCDs started once the SHCs got upgraded into HWCs. HWCs were not providing many services on rehabilitative care. Though HWCs showed a big improvement in meeting the curative care needs of people, many more services need to be added to realize the vision of comprehensive primary health care. It will require a bigger team in HWCs with an expanded range of skills. The global evidence has pointed towards the need for multi-disciplinary teams of health workers to cover the primary health care needs comprehensively [28].

HWCs showed important gains on the acceptability dimension. The people found the behaviour of HWC staff a lot more welcoming and supportive than what they had usually experienced in bigger public hospitals. This made it easier for the people to access the services at HWCs. The Mitanin community health workers were found to be important facilitators for people in using services of HWCs and this was in line with the policy expectations [11]. People visiting HWCs felt that the CHOs paid them enough attention and were responsive to their problems. This finding is in contrast to earlier evidence from PHCs in the neighbouring state of Maharashtra which had indicated strained relationship between the communities and the staff of PHCs [26]. Poor motivation level of PHC staff, especially the doctors, was found to be one of the key causes for that [26, 29].

The perceived cost barrier in accessing services at HWCs was found to be expectedly low. An earlier study had reported that the out-of-pocket expenditure on outpatient care in India was lowest in SHCs and PHCs among all government and private health facilities [30]. As the SHCs got developed into HWCs, they retained the cost advantage. The government policy prohibited any user fees to be charged in HWCs. The essential medicines and tests were mostly available and those were provided free by government. The shorter distance to HWCs also helped in reducing costs.

Another important finding was people’s satisfaction with the effectiveness of treatment and medicines they received from HWCs. But there were some unmet expectations. Some people expected HWCs to provide injections or intravenous drips, expecting immediate relief for acute problems. Such expectations of people seemed to be shaped by the practices of informal private providers in India. Earlier studies in India have highlighted that as one of reasons for the lower interest of people in services of PHCs [30]. HWCs will have to find ways to continue to attract more patients without resorting to such irrational care practices.

A study on PHC doctors in Maharashtra had found that they referred too many patients to higher centres [26, 29]. Excessive or unnecessary referrals had lowered the value of PHCs to people [29]. In case of HWCs, a conscious approach is to ‘refer less and resolve more’ [10, 25]. If HWCs refer everybody, why would anybody come to them [11]? Our study found that a judicious approach to referrals was being followed in HWCs and that was why people found the HWCs relevant. The approach of ‘refer less resolve more’ helped HWCs in gaining credibility among people. People did not complain of unnecessary referrals by the CHOs. But they reported other serious gaps in the referral system. Though the CHOs tried to guide the patients during referrals, going to higher facilities still involved severe difficulties for people including poor coordination, larger distance, incomplete care and higher costs. This shows the need for better systems for referral coordination from HWCs. Also, the availability of necessary services at secondary and tertiary facilities needs to be stronger for HWCs to become more useful to people.

The overall assessment of people regarding the quality of services at HWCs was largely positive. This finding is different from what the existing literature reports about the quality in Indian PHCs. A study had reported that the clinical quality of care in PHCs was weak, especially in the poorer states of the country [31]. A recent assessment of CHOs in HWCs has shown that their clinical competence was satisfactory for primary care, especially for NCDs such as hypertension and diabetes [32]. Investing in skills of CHOs has been recognised as the key to ensuring good quality services in HWCs [33].

The main aspects of quality that people found important included the effectiveness of care, staff behaviour and cleanliness in facilities. The people did not include the physical appearance of HWCs as an important aspect whereas the supply side i.e., the programme guidelines and monitoring system focused a lot on painting and branding of HWC buildings [6]. The people were also indifferent to the name given by government to HWCs. It may be useful for each state in India to decide a suitable name for its HWCs that is better in tune with the diversity of local cultures and languages. The use of digital technologies did not find much mention in the community feedback either, despite the widespread application of telemedicine and information technology at HWCs.

Another aspect missing in people’s feedback was regarding the Jan Arogya Samiti (JAS), the community-led committees being promoted by government to involve local people in functioning of HWCs [6]. A study had highlighted the positive role of initiatives on community action for health in improving the accountability of public sector health services in India [34]. Researchers have advocated for building community participation in governance and management of HWCs [11]. While this shows a gap in building vibrant community participation, the popularity HWCs enjoy among the communities suggests that it should be feasible to fulfil it if well directed efforts are made.

In terms of actionable policies to strengthen HWCs, our study recommends that all states in India need to ensure the following measures: (a) any HWC to be considered functional must have a CHO placed in addition to the pre-existing staff; (b) programmes should be institutionalised for continuous professional development for CHOs, aimed to meet primary health needs of people; (c) along with services for NCDs and acute ailments, primary care for injuries and mental illnesses should be made available through HWCs by improving the skills of CHOs, availability of necessary medicines and referral linkages; (d) in the long run, there should be plans to add more health workers to HWCs with the multi-disciplinary skills necessary to cover primary health care comprehensively; (e) systems need to be in place to ensure adequate and timely supply of essential medicines and point-of-care tests to HWCs; (f) an effective system needs to be set up for coordinating referrals from HWCs to higher facilities and back (g) the programme monitoring design should have emphasis on supervising those aspects of HWCs which are important to communities and patients e.g., the volume and range of health needs that HWCs address; (h) regular meetings of the community committees (JAS) need to be ensured by establishing facilitation mechanisms for community processes; (i) in line with the stated policy objective of making the services available within thirty minutes of walking distance for all people, additional HWCs should be approved in difficult geographies to cover the villages which are still too far from existing HWCs [6].

Earlier Indian literature had expressed the expectation that people would be willing to use the government primary care facilities when their services become adequately functional [10]. Our study confirms that to be the case with HWCs. People were happy to use the services of HWCs as they found the services relevant to their needs, easy to access and reasonably functional in terms of availability of providers and medicines. It is important to keep in mind that community expectations and perceptions of a health facility, such as HWCs, are highly dependent on their experience with what existed earlier i.e. the SHCs and PHCs. How communities assess the performance of HWCs may not always be reflective of the stated programme goals.

Limitations: Though the participants in FGDs were diverse in terms of age, sex and distance of residence from HWCs; their views cannot be assumed as representative of the rural population of the state. Most of the FGD participants were those who had tried to utilize the services of HWCs. Around half of the FGDs were conducted in HWC buildings and the participants there were mainly the patients visiting HWCs. The study sample was not stratified to ensure a substantive participation of individuals who had never used the services of HWCs.

## Conclusions

The community perspective indicates that the services of HWCs matched well with people’s needs. HWCs also added a significant value in improving accessibility, availability, acceptability, affordability and quality of curative primary care for the rural population. It shows that people are willing to use the public sector facilities for primary care when the services are relevant, accessible and of good quality. HWCs represent a shift from the selective to comprehensive primary health care in India and further expanding the range of services and skills of health workforce at HWCs alongside improving the referral pathways can bring the initiative closer to the lofty policy goals.

### Electronic supplementary material

Below is the link to the electronic supplementary material.

Supplementary Material 1

Supplementary Material 2

## Data Availability

The datasets used and/or analysed during the current study are available from the corresponding author and State Health Resource Centre, Chhattisgarh on reasonable request.

## Abbreviations

ANM: Auxiliary Nurse cum Midwife
CHW: Community Health Worker
HWC: Health and Wellness Centre
PHC: Primary Health Care
PHCs: Primary Health Centres
SHC: Sub Health Centre
UHC: Universal Health Coverage
