# Supplementary material for: How useful do communities find the health and wellness centres? A qualitative assessment of India’s new policy for primary health care
Source: BMC Prim Care. 2024 Mar 19;25:91. doi: 10.1186/s12875-024-02343-2 (PMC10949732; doi:10.1186/s12875-024-02343-2)
Supplement: Supplementary file 1 — Supplementary Material 1 [file 12875_2024_2343_MOESM1_ESM.docx]

**Additional File S1**

**Focus group discussion guide**

| **Name of the participant:** | **Age:**  Under 30 years/30-45 years/46-59 years/60 years |
| --- | --- |
| **Sex:** | **Caste group:**  SC/ST/OBC/Others |
| **Village:** | **HWC in the village:** Yes/No |

| **Sl.** | **Question** | **Probes** |
| --- | --- | --- |
|  | Are you aware of the presence of HWC? | What term do you use to refer to HWCs? Overall thoughts about the HWC, if any. |
|  | Do you visit the HWC for your health issues or for check-up? | In what kind of situations (health related), what kind of services [*facilitator starts a conservation using a recent experience with the HWC with one or two people*] (probe based on physical accessibility, availability of diagnostics, medicines etc., range of services, staff at HWC and interactions with them, role of Mitanins etc] |
|  | How has been your experience with the HWC services and the staff upon your visit? | Service availability, acceptability of services available at the facility, direct or indirect costs if any, experience with referrals etc. communication such as interactions with the staff, information being provided etc. |
| 4. | Do you think the services have improved from how they were before (compared with sub-health centre)? | If yes, how have they improved in terms of availability of diagnostics, medicines, preventive care, follow up, screening, referrals etc. If not, what do you think is missing? |
| 5. | What according to you are the strength of the healthcare services and healthcare providers in HWC? | In terms of expectations related to the accessibility of services (hours, proximity, informational etc.), availability of health services including preventive care, counselling, diagnostics, medicines, follow-up, delivery related care, emergency services etc.);behaviour, relationship with the health providers particularly CHOs; quality of services (cleanliness, approachability of providers, satisfaction with the care being provided etc). |
| 6. | What according to you are the weaknesses of the healthcare services and healthcare providers in HWC? | In terms of expectations related to the accessibility of services (hours, proximity, informational etc.), availability of health services including preventive care, counselling, diagnostics, medicines, follow-up, delivery related care, emergency services etc.) and behaviour, relationship with the health providers particularly CHOs. |
| 7. | Any suggestions that you would like to give. |  |
