# Supplementary material for: How useful do communities find the health and wellness centres? A qualitative assessment of India’s new policy for primary health care
Source: BMC Prim Care. 2024 Mar 19;25:91. doi: 10.1186/s12875-024-02343-2 (PMC10949732; doi:10.1186/s12875-024-02343-2)
Supplement: Supplementary file 2 — Supplementary Material 2 [file 12875_2024_2343_MOESM2_ESM.docx]

**Additional File S2**

**COREQ checklist**

Consolidated criteria for reporting qualitative studies (COREQ): 32-item checklist

Developed from: Allison Tong, Peter Sainsbury, Jonathan Craig, Consolidated criteria for reporting qualitative research (COREQ): a 32-item checklist for interviews and focus groups, International Journal for Quality in Health Care, Volume 19, Issue 6, December 2007, Pages 349–357, <https://doi.org/10.1093/intqhc/mzm042>

Please indicate in which section each item has been reported in your manuscript. If you do not feel an item applies to your manuscript, please enter N/A.

| **No.** | **Item** | **Description** | **Section #** |
| --- | --- | --- | --- |
| **Domain 1: Research team and reflexivity** | | | |
| Personal characteristics | | | |
| 1. | Interviewer/facilitator | Which author/s conducted the interview or  focus group? | SA conducted all the FGDs. |
| 2. | Credentials | What were the researcher's credentials? *E.g.*  *PhD, MD* | SA: MPH  SG: PhD  VRK: PhD |
| 3. | Occupation | What was their occupation at the time of the  study? | SA: Qualitative researcher  SG: Researcher and public health practitioner  VRK: Researcher and public health practitioner |
| 4. | Gender | Was the researcher male or female? | SA who conducted all the FGDs, was female. |
| 5. | Experience and  Training | What experience or training did the researcher  have? | SA, SG and VRK had experience with qualitative methods including facilitating focus groups and conducting interviews. |
| Relationship with participants | | | |
| 6. | Relationship  Established | Was a relationship established prior to study  commencement? | The research team did not have any contact with participants prior to obtaining informed consent. Researchers had no professional or ongoing relationship with the participants. |
| 7. | Participant knowledge of the interviewer | What did the participants know about the researcher? *E.g. Personal goals, reasons for*  *doing the research* | Participants were aware that this was a research project to explore the perceptions and experiences of community members with Health and Wellness Centres in Chhattisgarh as an information sheet about the study was shared with the participants. |
| 8. | Interviewer characteristics | What characteristics were reported about the interviewer/facilitator? *E.g. Bias, assumptions,*  *reasons and interests in the research topic* | The research team was interested in knowing the community perceptions and experiences related to the services provided in Health and Wellness Centres in Chhattisgarh. SG and VRK were facilitating the state government to strengthen the implementation of HWCs. |
| **Domain 2: Study design** | | | |
| Theoretical framework | | | |
| 9. | Methodological orientation and theory | What methodological orientation was stated to underpin the study? *E.g. grounded theory, discourse analysis, ethnography,*  *phenomenology, content analysis* | The research team conducted thematic analysis, and analyzed data both deductively and inductively. |
| Participant selection | | | |
| 10. | Sampling | How were participants selected? *E.g. purposive,*  *convenience, consecutive, snowball* | Purposive and convenience sampling was used to identify districts and participants respectively. |
| 11. | Method of approach | How were participants approached? *E.g. face-*  *to-face, telephone, mail, email* | Face-to-face. |
| 12. | Sample size | How many participants were in the study? | 75 |
| 13. | Non-participation | How many people refused to participate or  dropped out? What were the reasons for this? | 3. They were busy. |
| Setting | | | |
| 14. | Setting of data  collection | Where was the data collected? *E.g. home, clinic,*  *workplace* | Health and Wellness Centre (health facility) building, and residence of one of community members or Community Health Workers. |
| 15. | Presence of non-  participants | Was anyone else present besides the  participants and researchers? | Community Health Workers were present in a few FGDs conducted in the community. |
| 16. | Description of sample | What are the important characteristics of the  sample? *E.g. demographic data, date* | All participants were people from Chhattisgarh state. The average age of participants was 53 years. |
| Data collection | | | |
| 17. | Interview guide | Were questions, prompts, guides provided by  the authors? Was it pilot tested? | All questions, prompts and guides were provided by the authors. It was not pilot tested. |
| 18. | Repeat interviews | Were repeat interviews carried out? If yes, how  many? | None. |
| 19. | Audio/visual recording | Did the research use audio or visual recording  to collect the data? | Audio recordings were used. |
| 20. | Field notes | Were field notes made during and/or after the  interview or focus group? | Field notes were taken during the focus groups. |
| 21. | Duration | What was the duration of the interviews or  focus group? | The average duration of the focus group was 40 minutes. |
| 22. | Data saturation | Was data saturation discussed? | Data saturation was reached on almost all major topics. We didn’t reach saturation on the cultural  aspects from a patient’s perspective. |
| 23. | Transcripts returned | Were transcripts returned to participants for comment and/or correction? | Transcripts were not returned to participants for comment and/or correction. |
| **Domain 3: analysis and findings** | | | |
| Data analysis | | | |
| 24. | Number of data  coders | How many data coders coded the data? | One researcher coded the data. |
| 25. | Description of the  coding tree | Did authors provide a description of the coding  tree? | There is no description of the coding tree. |
| 26. | Derivation of themes | Were themes identified in advance or derived  from the data? | Themes were identified in advanced, sub-themes were derived from the data. |
| 27. | Software | What software, if applicable, was used to  manage the data? | Data was analyzed manually. |
| 28. | Participant checking | Did participants provide feedback on the  findings? | No participant feedback was taken. |
| Reporting | | | |
| 29. | Quotations presented | Were participant quotations presented to illustrate the themes / findings? Was each  quotation identified? *E.g. Participant number* | Key findings of this study were supported with  selected quotes in text. The quotes were identified. |
| 30. | Data and findings  consistent | Was there consistency between the data  presented and the findings? | All findings were derived from the data and all themes are supported by illustrative quotes. |
| 31. | Clarity of major  themes | Were major themes clearly presented in the  findings? | Major themes were informed by the Accessibility, Availability, Acceptability and Quality (AAAQ) framework. |
| 32. | Clarity of minor  themes | Is there a description of diverse cases or discussion of minor themes? | Yes, minor themes were emergent in nature. |
